# The hepatoprotective effects of fennel seeds extract and *trans*‐Anethole in streptozotocin‐induced liver injury in rats

**DOI:** 10.1002/fsn3.2090

**Published:** 2020-12-30

**Authors:** Zahra Samadi‐Noshahr, Mousa‐Al‐Reza Hadjzadeh, Reyhaneh Moradi‐Marjaneh, Abolfazl Khajavi‐Rad

**Affiliations:** ^1^ Student Research Committee Faculty of Medicine Mashhad University of Medical Sciences Mashhad Iran; ^2^ Department of Physiology Faculty of Medicine Mashhad University of Medical Sciences Mashhad Iran; ^3^ Neurogenic Inflammation Research Center Mashhad University of Medical Sciences Mashhad Iran

**Keywords:** Anethole, diabetic rats, fennel, liver injury

## Abstract

Hypoglycemic, anti‐inflammatory, and antioxidant activities of fennel have been recorded in numerous investigations. The study aimed to evaluate the protective effects of fennel or its active component *trans*‐Anethole (TA) on streptozotocin‐induced liver injury in rats. Rats were injected with a single dose of STZ (65 mg/kg) and treated with fennel (200 and 400 mg/kg), TA (80 mg/kg), or metformin (300 mg/kg) for 35 days. Serum lipid profile and liver enzyme activity (aminotransferases), oxidative stress markers, and the degree of fibrosis in the liver tissue were assessed. Both fennel and TA decreased blood glucose levels, reduced liver enzyme activity, food, and water intake, and intensity of weight loss, reduced serum triglycerides (TG), total cholesterol (TC), low‐density lipoprotein cholesterol (LDL‐c), and increased high‐density lipoprotein cholesterol (HDL‐c). Additionally, fennel and TA significantly reduced MDA concentration while increased CAT activity and thiol content and reduced the degree of injury and fibrosis in the liver of diabetic rats. Our results suggest that fennel seed extract and its active compound TA are able to protect the liver against diabetes‐induced hepatic injury in rats, probably via hypoglycemic and antioxidant effects.

## Graphical abstract

The effects of fennel seed extract and its active compound *trans*‐Anethole were investigated in the STZ‐induced liver injury in rats. Both fennel and *trans*‐Anethole effectively reduced blood glucose levels, food and water intake, and intensity of weight loss during the treatment. Also, both fennel and *trans*‐Anethole improved the serum lipid profile, decreased serum aminotransferases, attenuated liver tissue oxidative damage, and reduced liver fibrosis in diabetic rats.

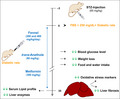

## 1 INTRODUCTION

Nonalcoholic fatty liver disease (NAFLD), including fatty liver, liver fibrosis, and cirrhosis, have been known as common consequences of diabetes (Lankarani et al., 2013; Bellentani et al., 2010). Many rodent models of type 1 and type 2 diabetes have been established to explain the pathogenesis of diabetes and to test novel therapies for the suppression of complications of diabetes (King, 2012; Liang et al., 2015). Streptozotocin (STZ), a glucosamine‐nitrosourea compound, is used to induce diabetes in experimental animals (Noshahr et al., 2020; Rahman et al., 2018). STZ by the induction of oxidative stress and lipid peroxidation damages the pancreatic beta cells and impairs insulin production (Ghasemi et al., 2014).

Oxidative stress occurs when the balance between the generation of reactive oxygen species (ROS) and antioxidant defense systems is impaired (Hosseinian et al., 2018). In diabetes, ROS is generated via different mechanisms and multiple sources, including the enzymatic pathway, nonenzymatic pathway, and also mitochondrial respiratory chain reactions (Palsamy et al., 2010). Hyperglycemia directly induces oxidative stress through the glycation of proteins, autoxidation of glucose, and the enhancement of the mitochondrial production of superoxide anion (Dias et al., 2005; Sheng et al., 2019). Moreover, increased levels of free fatty acids (FFA) in diabetes may also have a role in ROS production since mitochondrial uncoupling and β‐oxidation were increased (King & Loeken, 2004).

Biomarkers of oxidative stress have been reported to increase in the liver at the early stage of STZ‐induced diabetes due to its main role in detoxifying and oxidative processes (Schmatz et al., 2012). Furthermore, chronic hyperglycemia can induce the autoxidation of glucose, activation of protein kinase C, hexosamine metabolism, sorbitol formation, formation and glycation of methylglyoxal, and oxidative phosphorylation, which all are involved in the generation of ROS in the liver (Goswami & Chatterjee, 2014). Studies have shown that oxidative stress causes hepatic stellate cells (HSCs) to transform from quiescent to myofibroblast‐like cells, increasing the production of extracellular matrix proteins and inducing fibrosis (Vendemiale et al., 2001).

Fennel (*Foeniculum* V*ulgare* Mill.) is an aromatic plant, which is widely used as a food additive and for medicinal purposes (Malhotra, 2012). While all parts of the plant are important in the medicinal industry, fennel seeds are mostly used to extract essential oils (Malhotra, 2012). *trans*‐Anethole (TA), estragole, and fenchone are the main components of the fennel seed essential oils (Díaz‐Maroto et al., 2006). Fennel is a potent antioxidant (Goswami & Chatterjee, 2014) and also exerts anti‐inflammatory, anti‐bacterial, anti‐fungal, and anti‐parasitic effects (Asmat et al., 2016; Malhotra, 2012; Oktay et al., 2003). Studies have also reported hepatoprotective effects of fennel in various liver injuries (Ozbek et al., 2003; Parsaeyan, 2016).

TA [1‐Methoxy‐4‐(E)‐propenyl‐benzene] is an active compound present in the essential oil of fennel seeds (Fang et al., 2006). Previous studies have reported that TA exhibits various pharmacological activities, such as antioxidant, anti‐diabetic, cardioprotective, anti‐inflammatory, anti‐cancer, and estrogenic effects (Anitha et al., 2014; El‐Soud et al., 2011; He & Huang, 2011). In STZ‐induced diabetic rats, TA treatment has been reported to decrease blood glucose levels, increase insulin levels, and improve carbohydrate metabolic enzymes in the liver and kidneys (Sheikh et al., 2015). Considering the anti‐oxidative and anti‐diabetic effects of fennel and its main active compound, TA, the present study aimed to investigate the hepatoprotective effect of fennel and TA in STZ‐induced liver injury in rats.

## 2 MATERIAL AND METHODS

### 2.1 Chemicals

STZ (Sigma‐Aldrich Co.) was dissolved in 0.1 M citric buffer solution (pH: 4.2) and then immediately was used. Metformin (Sobhan Darou Co.) was dissolved in saline, and TA (Sigma‐Aldrich Co.) was dissolved in corn oil.

### 2.2 Animals

Thirty‐six male albino Wistar rats weighing 250 ± 30 g were supplied by the animal house of the Mashhad University of Medical Sciences. The animals were housed in a room with a 21 ± 2°C temperature and 12 hr light/dark cycle. All experimental procedures were in accordance with the guidelines approved by the Committee on Animal Research of Mashhad University of Medical Sciences, and the protocol and procedures employed were approved by the Research Ethical Committee of the Mashhad University of Medical Sciences (IR.MUMS.MEDICAL.REC.1398.202).

### 2.3 Collection of plant extraction

*Foeniculum vulgare* Mill. (Fennel) seeds were procured from the local market. The seeds were dried in the shade and stored at room temperature. The dried plant seeds were ground and then extracted with ethanol 70% (60–80°C) for 24 hr using the Soxhlet extraction assembly. After completion of extraction, the solvent was evaporated in a rotary evaporator, and then, the extract was transferred into a clean and dried vial and then stored at 4°C until use (Yazd et al., 2019).

### 2.4 Diabetes induction and treatment

Rats were randomized into six groups (*n* = 6/group) (Table 1). Diabetes induction was done by the intraperitoneal (i.p.) injection of STZ (65 mg/kg body) (Park et al., 2020). 72 hr after STZ injection, rats with fasting blood glucose (FBS) levels greater than 250 mg/dl and glycosuria were considered diabetic. Diabetic rats were treated with fennel extract (200 and 400 mg/kg) (Hassanpour et al., 2017), TA (80 mg/kg) (Sheikh et al., 2015) or metformin (300 mg/kg) (Ghatak et al., 2011) for 35 days. All rats were allowed free access to water and rat chow. The rats were monitored weekly for food and water intake and for body weight. Blood and urine samples were collected on the 0th, 21st, and 35th days.

**TABLE 1:** Experimental groups

Group	*n*	Injection	Treatment
Control	6	Saline solution	corn oil
STZ	6	STZ	corn oil
STZ‐TA	6	STZ	TA (80 mg/kg) dissolved in corn oil
STZ‐F200	6	STZ	Fennel (200 mg/kg) dissolved in corn oil
STZ‐F400	6	STZ	Fennel (400 mg/kg) dissolved in corn oil
STZ‐Met	6	STZ	Metformin (300 mg/kg) + corn oil

### 2.5 Sample collection

After 5 weeks of treatment, the rats were euthanized by anesthesia using the inhalant halothane. Fasting blood was collected from the heart, and then plasma was obtained. The liver tissues were removed, washed with ice‐cold saline, and then the two same regions of the liver right lobe were removed; a part was stored at −80°C until to use, and the other part was fixed at 10% buffered formalin for pathologic examination.

### 2.6 Histopathological examinations

The fixed tissues were embedded in paraffin wax by routine protocols and the provided sections (5 µm thick) were stained with hematoxylin and eosin (H&E) and Masson's Trichrome (Asia Pajhohesh) and examined using a photomicroscope (Olympus BX50, Japan).

### 2.7 Biochemical examinations

The animals underwent fasting, and then, blood samples were collected. The fasting blood glucose (FBG) levels, triglyceride (TG), total cholesterol, low‐density lipoprotein cholesterol (LDL‐c), and high‐density lipoprotein cholesterol (HDL‐c), alanine aminotransferase (ALT), and aspartate aminotransferase (AST) were measured in serum samples using commercially available kits and according the manufacturer's instructions (Pars Azmun, Iran). Urine volume and urine glucose levels were measured, and urinary glucose excretion rate was reported as g glucose/day.

### 2.8 Assessment of oxidative stress markers

#### 2.8.1 Malondialdehyde content

The concentration of malondialdehyde (MDA) was measured as an index of lipid peroxidation in the liver tissues. Briefly, the liver tissues were weighted and homogenized with ice‐cold phosphate buffer saline (PBS). 1 ml of the supernatant of homogenized samples was mixed with 1 ml of 10% trichloroacetic acid and 1 ml of 0.67% thiobarbituric acid. The samples were heated in a boiling water bath for 15 min and centrifuged at 1,000 *g* for 10 min. Then, the supernatants were collected, and the absorbance was read at 535 nm.

#### 2.8.2 Total thiol content

Liver tissue thiol content was measured using the Elmman's method described in previous works, in which the color development resulting from the reaction between the thiols within samples with DTNB (5, 5′‐dithiobis‐(2‐nitrobenzoic acid)) was measured at 412 nm.

#### 2.8.3 Assessment of SOD and CAT

The assessment of catalase (CAT) activity in the liver tissue homogenates was done by the method previously described by Aebi (Aebi, 1984). The method described by Madesh and Balasubramanian was used to determine the activity of superoxide dismutase (SOD) enzyme activity in the liver tissues (Madesh & Balasubramanian, 1998).

### 2.9 Statistical analysis

The sample size required for the experiment was estimated using G*power software (v3.1.9.7), considering FBS levels at the end of the study as a primary outcome based on our pilot study results. Considering statistical power = 0.95 and α = 0.05, the minimum sample size was estimated 5–6 animals per group. The analyses were carried out using SPSS software (version 16). One‐way ANOVA was run, and then, Tukey's post hoc test was used to compare the differences between the groups. All data were expressed as mean ± *SEM* *p* ≤ .05 was considered as a significant difference.

## 3 RESULTS

### 3.1 FBS, glucose excretion rate, and water intake

**FIGURE 1:**
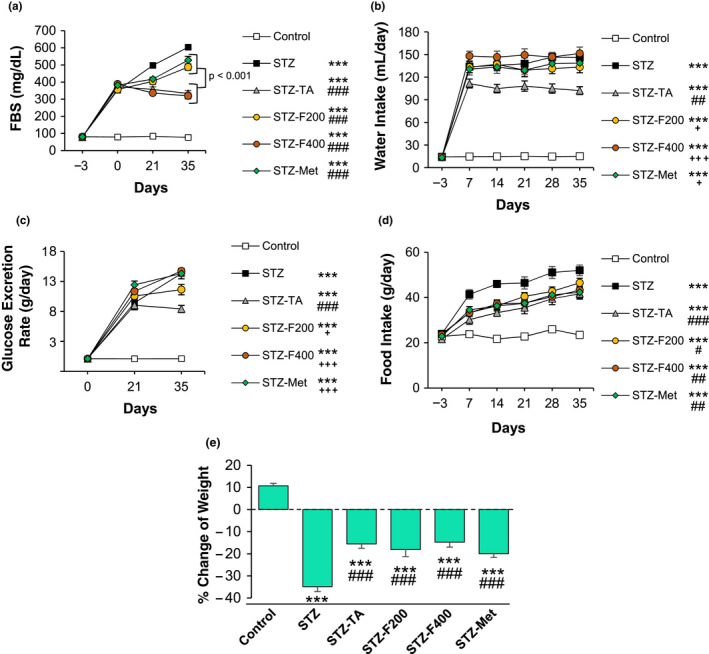
The effects of fennel and TA treatment on the (a) FBS, (b) water intake, (c) glucose excretion rate, (d) food intake, and (e) body weight changes of the STZ‐induced diabetic rats (*n* = 6). ***p < .001 shows the significant differences with the control group. ^#^ *p* < .05, ^##^ *p* < .01, ^###^ *p* < .001 shows the significant differences with the STZ group. ^+^ *p* < .05, ^++^ *p* < .01 shows the significant differences with the STZ‐TA group

All STZ‐injected rats developed severe hyperglycemia (>300 mg/dl) in the three days after STZ injection (day 0 of the experiment), and FBS level was gradually increased (up to 600 mg/dl) until the last day of the experiment in the nontreated rats compared to the control group (*p* < .001). In the STZ‐injected rats, which received oral administration of fennel (200 and 400 mg/kg), TA, or metformin FBS levels were significantly reduced (<400 mg/dl) (all, *p* < .001) compared to the STZ group (Figure 1a). TA and fennel 400 mg/kg were more effective in reducing the FBS levels compared to the fennel 200 mg/kg and metformin groups (*p* < .001) (Figure 1a). As expected, water intake in the STZ‐injected rats increased almost 15‐fold compared to the control animals (*p* < .001) (Figure 1b). Interestingly, while fennel treatment reduced the FBS levels, it did not reduce and even slightly increased water intake compared to the STZ group. On the other hand, TA significantly reduced water intake compared to the STZ group (*p* < .01) (Figure 1b). Glucose excretion rate (GER) was also investigated, and the results showed a significant increase in the GER in the STZ group compared to the control group (*p* < .001) (Figure 1c). Since TA significantly reduced FBS and water intake, glycosuria was also reduced in this group (*p* < .001), but while fennel reduced FBS, however, GER was not decreased in fennel‐treated rats compared to the STZ group (Figure 1c).

### 3.2 Weight gain and food intake

As shown in Figure 1d, food intake in diabetic rats was significantly increased compared to the control rats (*p* < .001). During the study, control rats gained weight; in contrast, diabetic rats lost as expected (*p* < .001) (Figure 1e). Treatment with TA, fennel (in both doses), and metformin significantly prevented weight loss during the study (all, *p* < .001) and reduced food intake compared to the STZ group (*p* < .05 ‐ *p* < .001); however, none of them reached to the control level. No significant differences were also observed between the treatment groups (Figure 1d,e).

### 3.3 Serum lipid profile and aminotransferase enzymes

The serum TC, TG, LDL‐c were significantly increased in diabetic rats (*p* < .001 for all), and HDL‐c was significantly decreased (*p* < .01). Additionally, aminotransferase enzymes (ALT and AST) were significantly higher in the STZ‐induced diabetic rats than in the control rats (*p* < .001). Treatment with fennel (200 and 400 mg/kg), TA, and metformin lowered the serum TG, TC, and LDL‐c levels and ALT and AST, while increased HDL‐c levels (*p* < .01‐*p* < .001) (Table 2).

**TABLE 2:** Effect of fennel and TA on serum lipid profile and hepatic enzymes in diabetic rats

Parameters	Groups					
	Control	STZ	STZ‐TA	STZ‐F200	STZ‐F400	STZ‐Met
TG (mg/dl)	65.83 ± 2.2^a^	223.83 ± 15.6^c^	126.1 ± 8.6^b^	144.1 ± 7.53^b^	131 ± 7.53^b^	137.67 ± 6.82^b^
TC (mg/dl)	102.3 ± 2.6^a^	193.3 ± 3.7^e^	131.3 ± 3^b^	154.3 ± 3.6^d^	134.3 ± 2.7^bc^	146.17 ± 2.2^cd^
LDL (mg/dl)	15.8 ± 0.7^a^	23.8 ± 0.94^b^	17.1 ± 0.7^a^	18.3 ± 1^a^	17.6 ± 0.76^a^	18.33 ± 0.76^a^
HDL (mg/dl)	36.8 ± 1.1^b^	28.8 ± 1.2^a^	37.6 ± 0.8^b^	39.5 ± 1.7^b^	38.8 ± 1.9^b^	38.4 ± 2^b^
AST (U/L)	130.5 ± 10.2^a^	434.8 ± 3.6^c^	271.6 ± 24.7^b^	323.5 ± 22.4^b^	280.3 ± 19.8^b^	265.6 ± 19.4^b^
ALT (U/L)	78.8 ± 4.46^a^	373.75 ± 17.5^c^	165.8 ± 11^b^	227 ± 18.4^b^	230.3 ± 26.4^b^	194.5 ± 10.82^b^

Note

Means with no letter in common in each raw are significantly different by Tukey's post hoc test at *α* = 5%. Data presented as means ± *SEM* (*n* = 6).

Abbreviations: ALT, alanine aminotransferase; AST, aspartate aminotransferase; HDL, high‐density lipoproteins; LDL, low‐density lipoproteins; TC, total cholesterol; TG, triglycerides.

### 3.4 Oxidative stress markers

**FIGURE 2:**
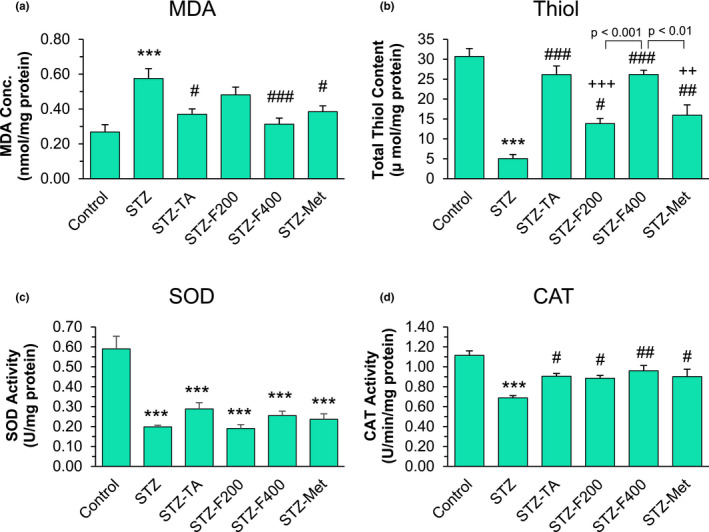
The effects of fennel and TA treatment on the (a) MDA, (b) total thiol content, (c) SOD and (d) CAT activities in the liver tissues of the STZ‐induced diabetic rats (*n* = 6). ***p < .001 shows the significant differences with the control group. ^#^ *p* < .05, ^##^ *p* < .01, ^###^ *p* < .001 shows the significant differences with the STZ group. ^+++^ *p* < .001 shows the significant differences with the STZ + TA group

As shown in Figure 2a, the concentration of MDA was significantly increased in the liver tissues of the diabetic rats than those of the control rats (*p* < .001). The MDA level in the diabetic rats treated with 400 mg/kg fennel, TA, as well as metformin was significantly decreased compared to the diabetic group (*p* < .001, *p* < .05, and *p* < .05, respectively), but it was not reduced to the control level (Figure 2a). The antioxidant system was also significantly weakened in the liver tissues of the nontreated diabetic rats, as evidenced by a decreased level of thiol content and activity of SOD and CAT enzymes (all, *p* < .001) (Figure 2b–d). Treatment of diabetic rats with fennel 200 and 400 mg/kg, TA and metformin significantly improved the total thiol content (*p* < .001, *p* < .05, *p* < .001 and *p* < .01, respectively). Both TA and fennel in 400 mg/kg doses were significantly more efficient in improving the thiol content of the liver when compared to the fennel 200 mg/kg and metformin groups (*p* < .001 and *p* < .01) (Figure 2b). In all treatment groups, SOD activity was significantly lower than the control group (*p* < .001), and although TA and fennel 400 mg/kg slightly increased SOD activity, but none of the treatments were able to significantly improve SOD activity compared to the STZ group. However, CAT activity was significantly improved in the STZ‐TA, STZ‐F200, STZ‐400, and STZ‐Met groups compared to the STZ group (*p* < .05, *p* < .05, *p* < .01, and *p* < .05, respectively) (Figure 2d).

### 3.5 Histopathological observation

**FIGURE 3:**
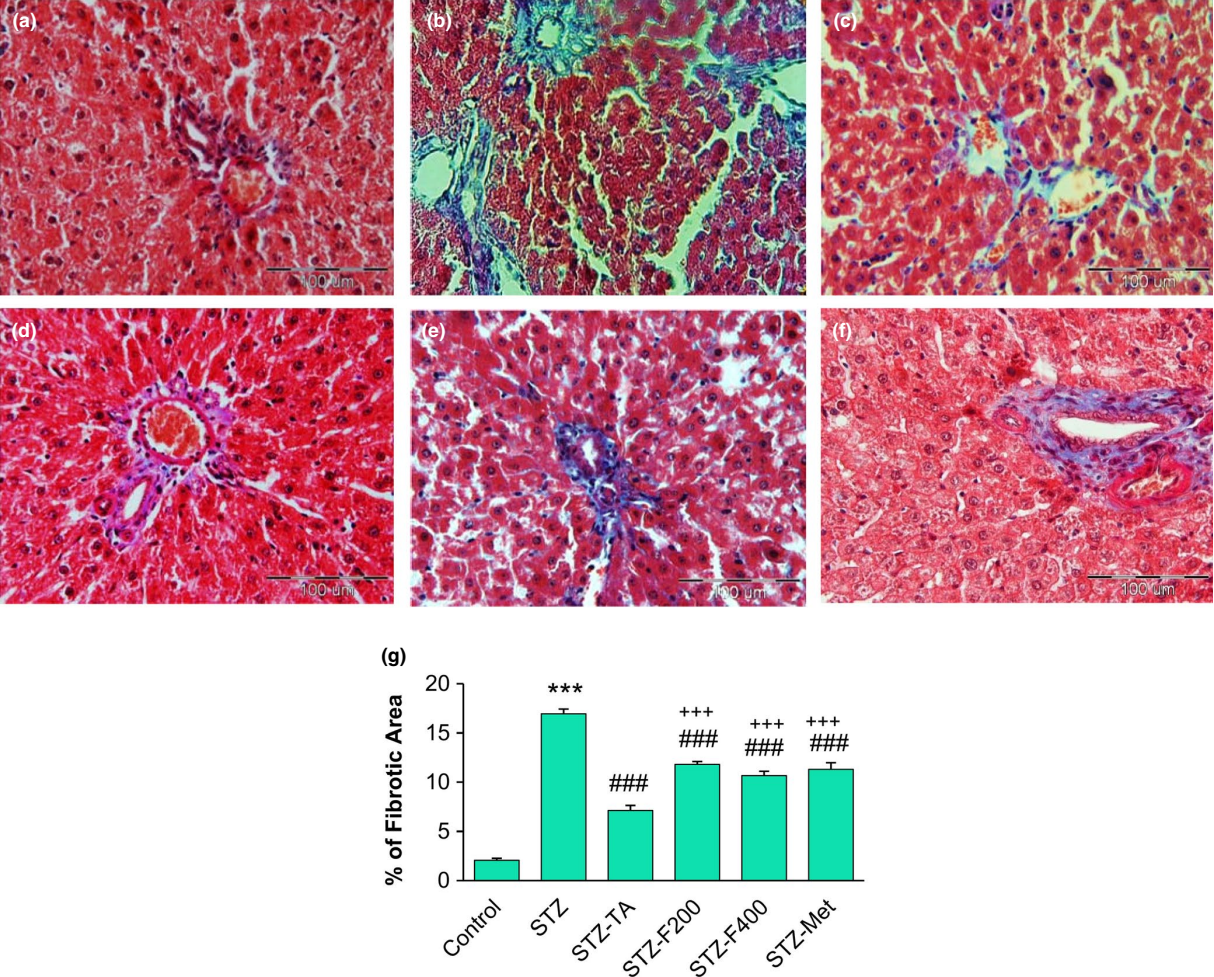
Treatment with fennel and TA attenuated collagen deposition in the liver of diabetic rats. Masson's trichrome stained images (×400), collagen stain in blue, which determine hepatic fibrosis, the nucleus in black, and cytoplasm pink or red (*n* = 6). (a): control group, (b): STZ group (c): STZ + TA 80 mg/kg, (d, e): STZ+(200 and 400 mg/kg fennel), (f): STZ + metformin and (g) The percentage of fibrotic area in liver tissue

Collagen content was determined by the image analyzer that measured the mean area percentage of collagen deposition. STZ‐injected rats showed a significant increase in collagen content around the portal vein compared to the control group (*p* < .001). In the TA, fennel (200 and 400 mg/kg), and metformin‐treated groups, hepatic fibrosis was significantly reduced compared with the STZ group (*p* < .001). TA was more effectively reduced fibrosis compared to fennel and metformin (*p* < .001) (Figure 3).

**FIGURE 4:**
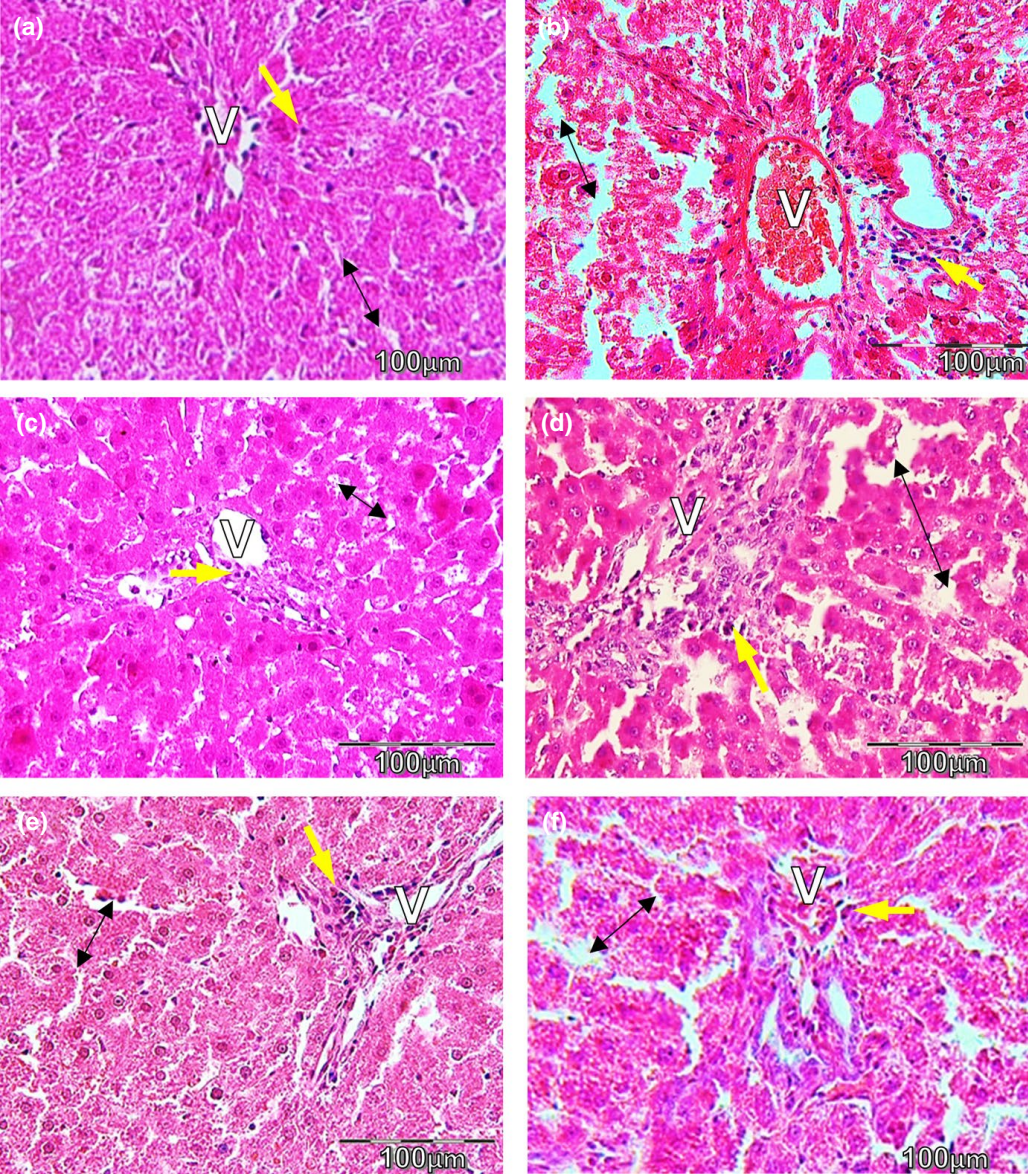
Treatment with TA and fennel attenuates liver damage in diabetic rats (H&E stain ×400), (a): control group, (b): STZ group (c): STZ + TA 80 mg/kg, (d, e): STZ+(200 and 400 mg/kg fennel), (f): STZ + metformin. (*n* = 6) Kupffer cells (arrow) shows infiltration of inflammatory cells; blood sinusoids (double arrow); and venous (V)

The histopathological findings of control rats revealed normal liver histology and structure (Figure 4a). Liver sections of the diabetic rats (Figure 4b) showed significant necrosis in most of the hepatocytes, increased monocyte infiltration, congested and dilated sinusoids, and thickening of the portal vein wall. Most hepatocytes of liver tissue from diabetic rats treated with TA (Figure 4c) appeared normal, but some signs of congested blood vein still were observed. Diabetic rats treated with fennel (200 mg/kg) showed improvement in some signs of hepatocyte necrosis and inflammation, but disarrangements of hepatocyte cords and dilated sinusoids were observed (Figure 4d). The liver tissue of diabetic rats treated with fennel (400 mg/kg) appeared normal histological structure and mild sinusoid dilation (Figure 4e). In diabetic rats treated with metformin, improvements in hepatocyte arrangement and monocyte infiltration were observed, but a congested and dilated portal vein was shown (Figure 4f).

## 4 DISCUSSION

The findings of the present study showed that in the STZ‐induced diabetic rats, morphological and biochemical alterations occurred in the liver tissue. Due to the STZ injection, serum glucose levels and glucose excretion rates were significantly increased, and therefore, water and food intake increased, and severe weight loss occurred. Treatment of diabetic rats with fennel, TA, or metformin reduced the blood glucose levels and the intensity of weight loss, normalized the serum lipid profile, and attenuated the liver damage and oxidative stress in the liver tissue of the diabetic rats.

Lack of insulin in the STZ‐injected rats led to severe weight loss and elevated serum glucose levels, consistent with previous reports (Vural et al., 2001). Fennel administration induced a potent reduction of FBS levels, food intake, and the intensity of weight loss compared to diabetic rats. Treatment with 400 mg/kg fennel and TA reduced FBS more significantly compared to metformin. Several mechanisms have been suggested for the blood‐glucose‐lowering effect of fennel, including effects on energy metabolism and increase of insulin secretion from remaining pancreatic cells (Anitha et al., 2014; El‐Soud et al., 2011). Abou El‐Soud et al. reported that fennel essential oil protects pancreatic β‐cells in STZ‐induced diabetic rats (El‐Soud et al., 2011). Moreover, Anitha et al. showed that the aqueous extract of fennel caused a significant decrease in FBS level by increasing serum insulin levels as well as hexokinase activity in renal and liver tissue (Anitha et al., 2014).

Despite the decrease in FBS levels in the fennel‐treated diabetic groups compared with the diabetic rats, water intake and urinary glucose excretion rate was still high and was not significantly different from the diabetic group. This indicates that a part of the hypoglycemic effect of fennel may be due to increased urinary excretion of glucose. Furthermore, in the TA‐treated diabetic rats, there was also a significant decrease in FBS levels. However, unlike fennel, TA was significantly water intake and reduced glucose excretion rate compared to the nontreated diabetic rats, as well as fennel and metformin‐treated diabetic rats. This may indicate that TA reduced blood glucose with a different path. Sheikh et al., 2015 suggested that the hyperglycemia ameliorating effect of TA is mediated by regulating the key enzymes involved in carbohydrate metabolism in STZ‐induced diabetic rats (Sheikh et al., 2015). Since TA is an active ingredient in fennel, a part of the beneficial effects of fennel extract in lowering blood glucose could be mediated through this mechanism.

Our findings also revealed that in the STZ‐induced diabetic rats, treatment with both fennel and TA significantly normalized the serum lipid profile by reducing the TG, TC, LDL‐c and increasing the HDL‐c levels. It has been reported that treatment with fennel extract leads to the recovery of the lipid profiles to normal levels in STZ‐induced diabetic rats (Anitha et al., 2014; Parsaeyan, 2016). These effects may be related to the lower blood glucose levels in treatment groups compared to the diabetic group. Additionally, fennel has been reported to increase the serum insulin levels and therefore normalize the serum lipid profile in diabetic rats (Anitha et al., 2014).

We determined the serum aminotransferase (ALT, AST) levels to evaluate the hepatic function. As shown in Table‐2, the AST and ALT levels were significantly increased in the STZ‐injected rats. AST and ALT are the cytosolic and mitochondrial enzymes of hepatocytes. The increase in serum ALT and AST levels may be due to the cellular damage in the liver caused by the direct toxic effect of STZ or hyperglycemia induced by it (Guven et al., 2006; Vural et al., 2001). In previous reports, a positive relationship between serum aminotransferase levels and the risk of diabetes has been reported in humans (Song et al., 2018). In line with our findings, also an elevated level of serum AST and ALT (3–4 fold) has been reported in the animal model of diabetes (Ramachandran & Saravanan, 2013; Zafar et al., 2009). In both fennel‐ and TA‐treated rats, aminotransferase levels were significantly reduced. As a result of liver cell damage and the disruption of the plasma membrane, AST and ALT are released and lead to the elevation of serum aminotransferase levels (McGill, 2016). We showed that fennel and TA attenuated hyperglycemia and resulted liver injury, as evidenced by the histopathological and biochemical assessments. Therefore, fennel and TA, maybe by the protection of the hepatocyte against hyperglycemia‐induced cell injury, were able to reduce liver enzyme levels in the serum.

In line with previous findings (Ramachandran & Saravanan, 2013), our results showed that STZ injection markedly induced oxidative stress in liver tissues as we showed a significant increase of MDA levels and a significant decrease in anti‐oxidative markers including total thiol content and SOD and CAT activities. While STZ mainly causes damage to pancreatic tissue, it also has direct toxic effects on other tissues such as the liver and kidney (Ghasemi et al., 2014). Single STZ injection has been reported to induce tissue damage and lipid peroxidation in mouse liver before hyperglycemia induction (Kume et al., 2004). Furthermore, hyperglycemia generates ROS in the enzymatic and nonenzymatic path, which in turn causes lipid peroxidation and hepatocyte damage (Asmat et al., 2016). Therefore, oxidative damage to the liver tissue could be induced by either the toxic effect of STZ or hyperglycemia or a combination of both or elucidated indirectly by other consequences of hyperglycemia such as tissue inflammation. However, we cannot exclusively determine the liver damages observed in this study arise from STZ direct toxicity, hyperglycemia, or other related factors. Nonetheless, extensive data are showing that high blood glucose induces liver damage in other diabetic models such as genetically modified diabetic animals, as well as in diabetic patients (Cordero‐Herrera et al., 2015; Garcia‐Compean et al., 2009; Mukai et al., 2006). Treatment with both fennel (400 mg/kg) and TA and metformin was able to significantly reduce lipid peroxidation compared to the diabetic group and restore the thiol content and CAT activity to normal levels. In line with these results, in STZ‐induced diabetes and chlorpyrifos‐induced liver injury models, treatment with fennel essential oil has been reported to exert anti‐oxidative effects in kidney and liver tissues (El‐Soud et al., 2011; Mansour et al., 2011). The antioxidant effects of fennel could be attributed to the hypoglycemic or presence of antioxidants, such as vitamins and phenolic compounds (Barros et al., 2009). Furthermore, oxidative stress in diabetic condition also can be triggered in liver by other mechanisms such as hyperglycemia‐induced tissue inflammation. Therefore, the anti‐oxidative effects of fennel could be applied through indirect pathways such as reducing inflammation, which is required more investigation in future studies.

Our histopathological findings showed that fennel and TA reduced liver damage and dysfunction in diabetic rats. It seems that fennel and TA reduced hepatic tissue inflammation by inhibiting neutrophilic accumulation and the generation of free radicals. The influx of fatty acids into hepatocytes due to hyperinsulinemia in diabetes is more than the capacity of excretion of protein, such as apolipoprotein B of hepatocytes. This change is indicative of the formation of fatty liver (Mantena et al., 2008; Remedio et al., 2011). We also detected that STZ injection led to perisinusoidal fibrosis and increased collagenous material. Five‐week treatment with TA and fennel (in both doses) reduced the fibrotic area. TA more effectively reduced hepatic fibrosis compared to fennel and metformin. The anti‐fibrotic effects of TA, in addition to the hypoglycemic effect, also maybe are related to the estrogen‐like properties (Albert‐Puleo, 1980; Marinov & Valcheva‐Kuzmanova, 2015). There is increasing clinical and experimental evidence indicating that estrogen plays a role in glucose homeostasis and reduces the complications of diabetes, such as fibrosis (Bissell, 1999; Mankhey et al., 2005; Maric et al., 2004; Yasuda et al., 1999). TA has been reported to exert estrogenic activity (Dhar, 1995), and therefore, it is possible that a part of the anti‐diabetic effects of TA may arise from its estrogenic activity; however, more studies are needed to confirm this hypothesis. Moreover, we have not investigated the exclusive markers of fibrosis such as collagen I, fibronectin, and α‐SMA, and therefore, further studies are needed to determine the exact anti‐fibrotic mechanism of fennel and TA.

## 5 CONCLUSION

Our findings indicated that the administration of fennel or its active component TA is able to protect the liver against STZ‐induced diabetes in rats. They are also able to ameliorate blood glucose levels and normalize lipid profile in serum. A part of the therapeutic effects of fennel extract and TA is possibly through the hypoglycemic properties of them. Additionally, they may directly protect the liver through other mechanisms such as antioxidant effects. Overall, these findings suggest that fennel or its active compound, TA, could be useful in the management of diabetes complications.

## CONFLICT OF INTEREST

The authors declare that they do not have any conflict of interest.

## ETHICAL APPROVAL

This study was approved by the Research Ethical Committee of the Mashhad University of Medical Sciences (IR.MUMS.MEDICAL.REC.1398.202).
